# Epidemiological study of strongyle infections in equines in Egypt: prevalence and risk factors

**DOI:** 10.3389/fvets.2026.1901312

**Published:** 2026-08-19

**Authors:** Abdelfattah Selim, Mohamed Marzok, Hattan S. Gattan, Mohammed H. Alruhaili, Hesham Ismail, Adel I. Almubarak, Abdelrahman M. Hereba

**Affiliations:** 1 Department of Animal Medicine (Infectious Diseases), Faculty of Veterinary Medicine, Benha University, Toukh, Egypt; 2 Department of Clinical Sciences, College of Veterinary Medicine, King Faisal University, Al-Ahsa, Saudi Arabia; 3 Department of Medical Laboratory Sciences, Faculty of Applied Medical Sciences, King Abdulaziz University, Jeddah, Saudi Arabia; 4 Special Infectious Agents Unit, King Fahad Medical Research Center, King AbdulAziz University, Jeddah, Saudi Arabia; 5 Department of Clinical Microbiology and Immunology, Faculty of Medicine, King AbdulAziz University, Jeddah, Saudi Arabia; 6 Department of Public Health, College of Veterinary Medicine, King Faisal University, Al-Ahsa, Saudi Arabia; 7 Department of Microbiology, College of Veterinary Medicine, King Faisal University, Al-Ahsa, Saudi Arabia

**Keywords:** donkey, horse, Nile Delta, risk factor, risk factors, strongylosis

## Abstract

**Background:**

Equine strongylosis is one of the most prevalent gastrointestinal parasitic diseases affecting horses and donkeys worldwide, leading to reduced health, productivity, and working performance. This study aimed to determine the prevalence of gastrointestinal strongyle infection and identify associated risk factors among equines in three Egyptian governorates.

**Methods:**

A cross-sectional study was conducted on 540 horses and donkeys. Fresh fecal samples were collected and examined using the magnesium sulfate flotation technique for the detection of strongyle eggs. Associations between infection and potential risk factors were evaluated using chi-square analysis, followed by multivariable logistic regression to identify independent predictors of infection.

**Results:**

The overall prevalence of gastrointestinal strongyle infection was 39% (214/550). Horses showed a slightly higher prevalence (40%) than donkeys (37%). Infection was significantly associated with sex, age, management system, body condition score, and deworming history (*P* < 0.05). Multivariable logistic regression identified male sex (OR = 2.8, 95% CI: 1.7–4.4), age <2 years (OR = 3.1, 95% CI: 1.8–5.2), grazing management (OR = 3.4, 95% CI: 1.8–6.6), poor body condition (OR = 2.8, 95% CI: 1.3–5.9), and absence of regular deworming (OR = 4.1, 95% CI: 2.4–7.1) as significant independent risk factors for strongyle infection.

**Conclusion:**

Gastrointestinal strongyle infection is common among Egyptian equines and is associated with several management- and host-related factors. Improving grazing management, implementing strategic deworming programs, and enhancing owner awareness are likely to reduce infection risk and improve equine health and productivity. Future studies incorporating quantitative fecal egg counts and molecular characterization are recommended to better assess infection intensity and parasite diversity.

## 1 Introduction

Equine internal parasites are generally classified into three main groups based on their morphology: nematodes (roundworms), cestodes (tapeworms), and trematodes (flukes) (1). Each group exhibits distinct structural characteristics as well as unique growth patterns and life cycles. Among these, roundworms (nematodes) are considered the most economically significant internal parasites affecting equines (2, 3).

The severity of damage caused by these parasites varies depending on the parasite species, their load, and the nutritional and immune status of the equids. They negatively affect animal performance, production, and productivity, primarily through weight loss, poor weight gain, and, in severe cases, can even lead to increased mortality (4–6).

Equines are susceptible to a wide range of internal parasites, including roundworms—particularly strongyles—as well as flukes, tapeworms, protozoa, and fly larvae. These parasites can cause significant damage to the intestinal tract, respiratory system, and various internal organs of the host (7–10). Strongyle nematodes are significant gastrointestinal parasites of equines, classified under the Superfamily Strongyloidea, Family Strongylidae, and Genus *Strongylus*, which includes three main species: *Strongylus vulgaris, Strongylus edentatus*, and *Strongylus equinus*. These parasites reside as adults in the large intestine of equids (5, 11).

Equine strongyles are classified into large and small strongyles, with the large ones being more pathogenic than the smaller species. Strongylosis poses a serious health threat, especially among young horses raised on permanent pastures. However, severe infections can also occur in adult horses, particularly under conditions of overcrowding and poor management (12, 13). Among the Strongylus species, *S. vulgaris* is considered the most pathogenic and is commonly referred to as the double-toothed strongyle, while *S. edentatus* is known as the toothless strongyle, and *S. equinus* is called the triple-toothed strongyle. Comparatively, *S. vulgaris* is smaller in size than *S. edentatus* (11, 14, 15).

In Egypt, *Strongylus* spp. are among the most prevalent gastrointestinal nematodes affecting equines, particularly in regions with inadequate parasite control programs. Several studies have reported infection rates exceeding 60% in horses and donkeys (16–18). The infestation is widespread in both rural and peri-urban areas, where grazing management is often suboptimal and deworming practices are irregular (19). These parasitic burdens result in substantial economic losses through decreased productivity, higher treatment costs, and lower market value of affected animals, especially in communities dependent on equines for labor, transportation, and income generation (20, 21).

Therefore, this study aimed to determine the prevalence of strongyle infections in horses and donkeys across three Egyptian governorates and to identify the risk factors associated with the disease.

## 2 Materials and methods

### 2.1 Ethical statement

The study was approved by the Ethics Committee of the Faculty of Veterinary Medicine, Benha University, Egypt. All procedures involving animals were conducted in accordance with the ethical guidelines and regulations set forth by the committee (ethical Approval FVTM02-04-2023). Prior to sample collection, verbal informed consent was obtained from the animal owners after explaining the purpose and procedures of the study.

### 2.2 Description of the study area

**Figure 1:**
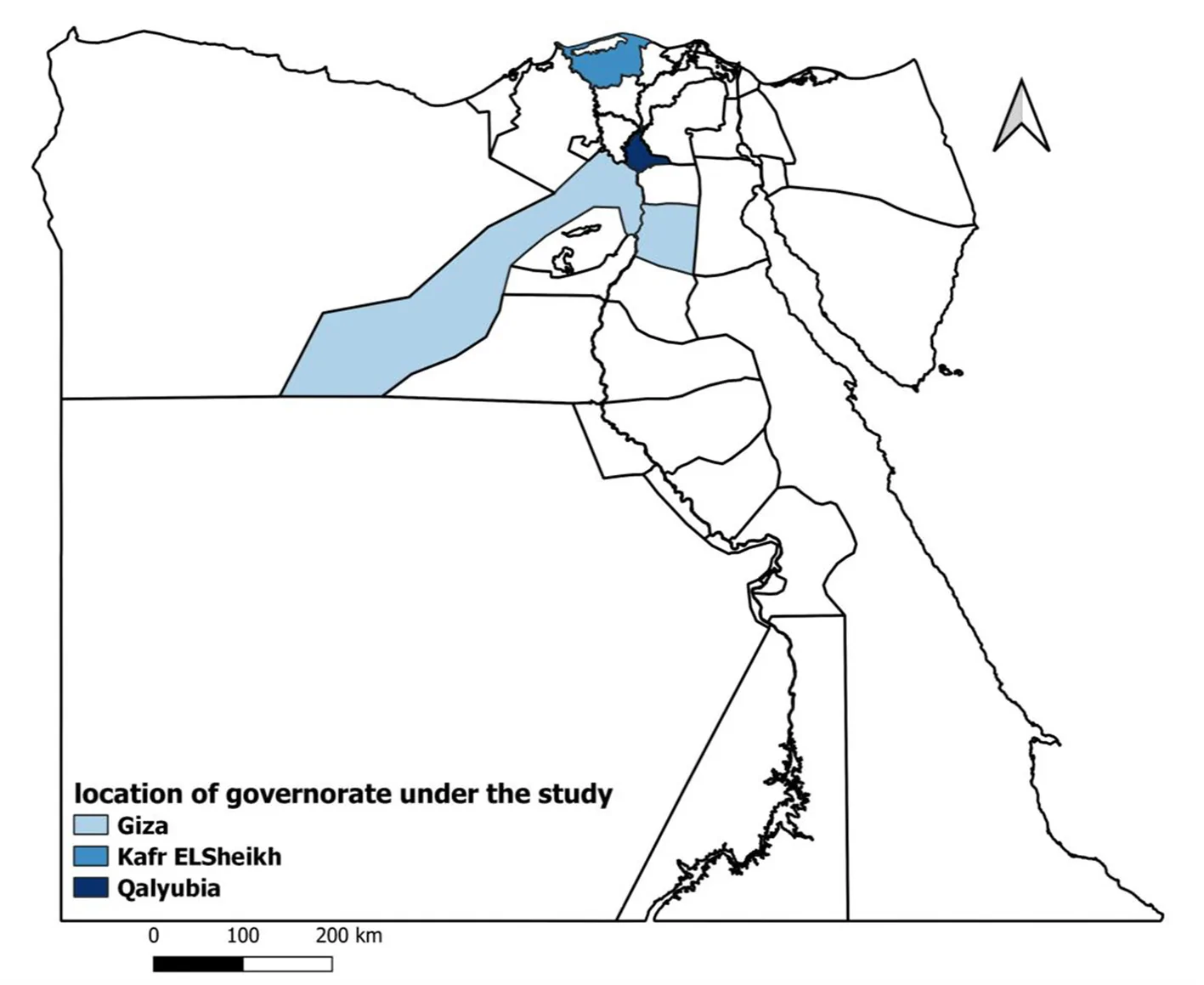
Geographic distribution of Giza, Kafr ElSheikh and Qalyubia in Egypt (map created with QGIS).

A cross-sectional study was conducted on equines across three Egyptian governorates (Giza, Kafr El-Sheikh, and Qalyubia) from January to December 2024. These governorates are located in the northern part of Egypt, within the Nile Delta and surrounding regions (Figure 1). Giza, part of Greater Cairo, lies to the west of the Nile and is characterized by peri-urban agricultural zones and rural communities where equines are commonly used for transportation and farm labor. Kafr El-Sheikh, situated in the north-central Delta and bordering the Mediterranean Sea, has a predominantly agricultural economy where donkeys and horses are integral to daily farming activities, particularly in land cultivation and produce transport. Qalyubia, just north of Cairo, is a densely populated governorate with a mixed urban-rural setting; here, equines play a vital role in small-scale agriculture, brick kilns, and local transportation, especially in areas inaccessible to motorized vehicles.

The climate in all three governorates is classified as Mediterranean arid, with mild, wet winters and hot, dry summers. Average temperatures range from 9–18 °C in winter to 25–38 °C in summer, and rainfall is sparse, typically occurring between November and March. These environmental conditions, combined with variable pasture hygiene and limited veterinary infrastructure in rural zones, may influence the transmission dynamics and persistence of parasitic infections such as strongylosis among equine populations.

### 2.3 Sampling and sample size

A cross-sectional study was conducted between January and December 2024 to estimate the prevalence of strongyle infections in equines across three Egyptian governorates. The required sample size was calculated using the formula described by Thrusfield (22):

where:

- *N* is the required sample size,
- *1.96* is the *Z*-value corresponding to a 95% confidence level,
- *Pexp* is the expected prevalence (assumed at 50% due to lack of precise previous data),
- *d* is the desired absolute precision (set at 5%).

Using these parameters-−95% confidence level, 5% precision, and 50% expected prevalence—the minimum sample size was calculated to be 384. However, to increase the study's statistical power and account for potential data loss or variability across the study regions, a total of 550 fecal samples were collected from horses and donkeys in Giza, Kafr El-Sheikh, and Qalyubia governorates. Sampling was conducted randomly from both privately owned and working equines in rural and peri-urban areas, ensuring adequate representation of different local management systems and environmental conditions.

### 2.4 Sample collection and handling

Using gloved hands, approximately 25 grams of fecal material was collected directly from the rectum of restrained equines at each study site. Samples were deposited into screw-capped universal sample bottles to ensure secure storage and prevent contamination. Each sample was uniquely labeled with the corresponding animal's identification data, including species (horse and donkey), age (< 2, 2–4, >4 years), sex (male and female), sampling site, body condition score (poor, medium, and good), management type (grazing, stable, and mixing), and deworming status (Yes and No).

### 2.5 Parasitological techniques

To isolate parasite eggs from fecal debris, the flotation technique was employed. Approximately 3 g of fecal material was placed into a clean glass beaker and mixed with 40 mL of magnesium sulfate flotation solution (specific gravity = 1.28). The mixture was thoroughly stirred using a glass rod until a homogeneous suspension was obtained and then filtered through a tea strainer into another beaker to remove coarse debris. The filtered suspension was transferred into a test tube until a positive meniscus formed, and a coverslip was carefully placed on the top of the tube. After standing undisturbed for 20 min, the coverslip was gently removed, placed on a clean microscope slide, and examined initially using a 10 × objective lens (100 × total magnification) for the detection of parasite eggs. Suspected eggs were further examined under higher magnification when necessary for confirmation. Strongyle eggs were identified based on their distinct oval morphology, following the criteria described by William (23).

### 2.6 Statistical analysis

All relevant data were collected, coded, and accurately entered into Microsoft Excel before being exported to IBM SPSS Statistics for Windows, Version 24.0 (IBM Corp., Armonk, NY, USA) for statistical analysis. Descriptive statistics were applied to determine the prevalence and corresponding percentages of strongyle infections among the sampled equines.

To evaluate the association between strongyle infection and potential risk factors, the Pearson chi-square test (χ^2^) was used. Additionally, both bivariate and multivariate logistic regression analyses were performed to quantify the strength of association between infection status and identified risk variables. The results were reported using odds ratios (OR), 95% confidence intervals (CI), and a *P*-value threshold of < 0.05 to determine statistical significance.

## 3 Results

Out of the 550 animals examined, 214 (39%) tested positive for gastrointestinal equine strongyles in both species (Table 1). Among these positive cases, 67 (approximately 31.3%) were donkeys and 147 (68.7%) were horses. Additionally, the study revealed that parasitic burdens were slightly higher in males (48%) compared to females (21%), though the difference was statistically significant (< 0.0001), Table 1.

**Table 1:** Prevalence of strongyle infections in equines from three Egyptian governorates in associations with different variables.

Variable	Total tested horses	No of positive	% of positive	95% CI	Statistic
Locality				
Giza	180	63	35	28.41–42.22	χ^2^ = 3.667 df = 2 *P* = 0.160
Kafr ElSheikh	190	71	37	30.81–44.44	
Qalyubia	180	80	44	37.37–51.74	
Sex				
Male	364	175	48	42.99–53.21	χ^2^ = 38.059 df = 1 *P* = < 0.0001^*^
Female	186	39	21	15.74–27.38	
Species				
Horse	367	147	40	35.16–45.14	χ^2^ = 0.609 df = 1 *P* = 0.435
Donkey	183	67	37	29.96–43.8	
Age				
< 2 years	210	111	53	46.12–59.5	χ^2^ = 30.504 df = 2 *P* < 0.0001^*^
2–4 years	187	64	34	27.8–41.28	
>4 years	153	39	25	19.24–32.94	
Management				
Grazing	267	145	54	48.32–60.18	χ^2^ = 56.088 df = 2 *P* < 0.0001^*^
Stable	184	53	29	22.74–35.72	
Mixed	99	16	16	10.2–24.65	
Body condition				
Poor	281	159	57	50.73–62.25	χ^2^ = 78.909 df = 2 *P* < 0.0001^*^
Medium	176	43	24	18.67–31.28	
Good	93	12	13	15.27–32.07	
Deworming status				
No	392	192	49	44.07–53.91	χ^2^ = 58.219 df = 1 *P* < 0.0001^*^
Yes	158	22	14	9.38–20.18	
Total	550	214	39	34.93–43.05	

^*^Results were considered statistically significant at *P* < 0.05.

In addition, the prevalence of strongyle infection was significantly higher (*P* < 0.05) among young equines under 2 years of age (53%), those managed under grazing systems (54%), animals in poor body condition (57%), and those with no history of deworming (49%; Table 1).

To evaluate the simultaneous influence of all associated factors on strongyle infection, a multivariate logistic regression analysis was conducted, using a 95% confidence interval and a significance level of *P* < 0.05 (Table 2). Odds ratios (OR) were used to quantify the strength of association between each risk factor and strongyle infection. The analysis showed that male equines were more likely to be infected (OR = 2.8, 95% CI: 1.7–4.4). Additionally, the risk of infection was significantly higher in animals under 2 years of age (OR = 3.1, 95% CI: 1.8–5.2), those managed under grazing systems (OR = 3.4, 95% CI: 1.8–6.6), animals in poor body condition (OR = 2.8, 95% CI: 1.3–5.9), and those with no history of deworming (OR = 4.1, 95% CI: 2.4–7.1; Table 2).

**Table 2:** Multivariate logistic regression analysis of risk factors associated with strongyle infections in equines.

Variables	*B*	SE	OR	95% CI for OR		*P* value
				Lower	Upper	
Sex						
Males	1.017	0.239	2.8	1.7	4.4	< 0.0001
Age						
< 2 years	1.124	0.265	3.1	1.8	5.2	0.000
2–4 years	0.239	0.272	1.3	0.7	2.2	0.037
Management						
Grazing	1.227	0.335	3.4	1.8	6.6	< 0.0001
Stable	0.343	0.350	1.4	0.7	2.8	0.032
Body condition						
Poor	1.032	0.375	2.8	1.3	5.9	0.006
Medium	0.814	0.387	2.3	1.1	4.8	0.036
Deworming						
No	1.413	0.279	4.1	2.4	7.1	< 0.0001

B, Logistic regression coefficient; SE, standard error; OR, odds ratio; CI, confidence interval.

## 4 Discussion

Gastrointestinal (GI) parasitic infections directly impact the health and productivity of working equines, leading to decreased work performance and, consequently, reduced income for both their owners and the surrounding community (24). In the present study, the overall prevalence of strongyle infection among horses and donkeys was estimated at 39% (214/550), based on coprological microscopic examination across three Egyptian governorates.

The prevalence reported in the current study was lower than several previously published figures. For example, Enigidaw et al. (25) documented a prevalence of 47.4% in horses and donkeys in Ethiopia, while Negash et al. (14) reported a higher rate of 53.13% in northern Ethiopia. Similarly, Pandey and Eysker (26) found a prevalence of 48% in donkeys in Zimbabwe. In addition, Mangassa and Tafese (5) reported a prevalence of 46.1% in and around Batu Town, East Shewa Zone, Oromia Regional State, Ethiopia. Notably, Bogale et al. (27) recorded an even higher rate of 84.77%. Interestingly, the prevalence of strongyle infections reported in the current study was higher than previously recorded rates in other regions of Egypt, such as Luxor and the governorates of Al-Dakahlia and Al-Sharqia, where prevalence rates were 9% (28) and 11.6% (29), respectively.

These discrepancies could be explained by variances in parasite epidemiology, management techniques, irregular deworming programs, the lack of specialized donkey sanctuaries, and pasture grazing limits (6, 15, 27, 30–32).

The present findings revealed that the prevalence of strongyles infections was higher in horses (40%) than donkeys (37%), which come in agreement with findings of Negash et al. (14).

However, the present study reported a higher species-level prevalence compared to the findings of Disassa et al. (33), who recorded rates of 5.82% in donkeys and 4.92% in horses in and around Dangila town. Conversely, the prevalence observed in this study was lower than that reported by Hassan et al. (34), who documented a prevalence of 99.15% in Sudan. The observed disparities could be the result of variances in parasite load, and agroecological circumstances, access to routine deworming services, and hygiene managment (31, 32, 35–37).

The present study estimated the prevalence of equine strongylosis at 21% in females and 48% in males, with a statistically significant difference (*P* < 0.0001). Similar findings were reported by Molla et al. (38) and Bogale et al. (27), who also observed a significant association between parasitic infection and sex. This disparity may be explained by management practices, as males are mainly use for drafting or transport, whereas females are frequently kept at for breeding purposes. This may expose males to higher levels of strongyle infection than females (1). Nonetheless, Fikru et al. (39) found no significant association between sex and parasite infection. This could be due to a number of variables, including biological differences, access to anthelmintic treatment, changes in husbandry techniques, and communal grazing systems (14, 36, 40–42).

Statistical analysis revealed a significant association between age and strongyle infection (*P* < 0.05). A similar trend was reported by Mangassa and Tafese (5), who observed prevalence rates of 62.86%, 41.42%, and 37.23% in young, adult, and old equines, respectively. However, these findings contrast with those of Sultan et al. (43), who reported prevalence rates of 25.7% in young, 61% in adults, and 13.2% in older animals. The higher infection rate in younger equines may be attributed to their underdeveloped immune systems (44, 45). Conversely, Ibrahim et al. (46) reported a statistically significant difference among age groups, with a higher prevalence observed in adults and a lower rate in young animals.

Young animals are more susceptible to strongyle infections because their immune system is still immature, making it harder to mount an effective defense (47, 48). The Th2 immune response—crucial for combating gastrointestinal nematodes—is not yet fully developed, leading to weaker cytokine activity and reduced activation of protective cells like eosinophils. Strongyles also take advantage of this developmental stage by modulating or evading host immunity, allowing easier establishment and higher parasite burdens. Together, immature immunity, an underdeveloped Th2 response, and host-parasite interactions explain the increased vulnerability of young animals (49).

Management type was identified as a major factor influencing the variation in strongyle parasite prevalence (*P* < 0.0001). Prevalence rates of 54%, 29%, and 16% were recorded among animals under grazing, stable, and mixed management systems, respectively. These findings are lower than those reported by Mezgebu et al. (50) who documented prevalence rates of 97.14%, 80.43%, and 83.33% for animals fed on pasture, mixed, and grain-based systems, respectively. This discrepancy may be attributed to differences in climatic conditions and seasonal variations between the study locations (41, 42, 51, 52).

The current study also revealed a statistically significant association (*P* < 0.0001) between body condition score and strongyle infection, with prevalence rates of 57%, 24%, and 13% in animals with poor, medium, and good body conditions, respectively. These results align with the findings of Mangassa and Tafese (5), who reported prevalence rates of 59.26%, 43.17%, and 30.6% for poor, medium, and good body conditions, respectively, demonstrating a similar trend. However, the infection rates observed in the current study were lower than those reported by Tesfu et al. (53), who recorded higher prevalence rates of 72.5%, 71.6%, and 70.7% in poor, medium, and good body condition scores, respectively. Strongyles infection is higher in equines with poor body condition because weakened immunity due to malnutrition reduces their ability to resist parasitic infestations. Additionally, chronic strongyle infection itself contributes to weight loss and poor condition, creating a cycle of increased susceptibility and deteriorating health (54–58).

The history of deworming was identified as a significant risk factor influencing the prevalence of strongyle infection (*P* < 0.0001), with a higher prevalence observed in non-dewormed animals, consistent with the findings of Mangassa and Tafese (5). The presence of infection in dewormed equines may be attributed to the temporary suppression of egg production by adult worms rather than complete elimination, or to the development of anthelmintic resistance. Additionally, the use of low-quality or substandard anthelmintics may contribute to treatment failure (17). Moreover, the absence of eggs during examination may be explained by the prepatent period of strongyles, which can last 5–7 months (59), or due to hypobiosis, where larvae become arrested in development during unfavorable environmental conditions.

This study focused solely on determining the prevalence of strongyle infection based on the presence or absence of eggs in fecal samples. A key limitation is the absence of a quantitative diagnostic technique, such as the McMaster method, which would have allowed for the calculation of eggs per gram (EPG). Without this data, it is not possible to assess the intensity or severity of infection (i.e., light, moderate, or heavy), which limits the depth of interpretation regarding the clinical or epidemiological significance of the findings.

## 5 Conclusion

This study highlights the significant impact of strongyle infections on the health and productivity of equines in Egypt, with an overall prevalence of 39% across three governorates. The findings revealed significant associations between strongyle prevalence and various risk factors, including sex, age, body condition, management practices, and deworming history. Higher infection rates were notably observed in males, young animals, poorly conditioned equines, and those under grazing systems or lacking regular deworming, underscoring the role of environmental exposure and inadequate parasite control measures. The observed variations emphasize the importance of integrated parasite control strategies, including improved management practices, routine and effective deworming programs. Enhancing awareness among owners and improving veterinary services particularly the quality and monitoring of anthelmintic treatments will be crucial to reducing infection rates and improving equine welfare and productivity in Egypt.

## Data Availability

The original contributions presented in the study are included in the article/supplementary material, further inquiries can be directed to the corresponding author.
