# Correction: Epidemiological study of strongyle infections in equines in Egypt: prevalence and risk factors

**DOI:** 10.3389/fvets.2026.1974891

**Published:** 2026-08-26

**Authors:** Frontiers Production Office

**Affiliations:** Frontiers Media SA, Lausanne, Switzerland

**Keywords:** donkey, horse, Nile Delta, risk factor, risk factors, strongylosis

The funder Deanship of Scientific Research (DSR), Proposal number KFU263777, was erroneously omitted from the original article. The correct Funding Statement reads:

The author(s) declared that financial support was received for this work and/or its publication. The project was funded by KAU Endowment (WAQF) at king Abdulaziz University, Jeddah, Saudi Arabia. The authors, therefore, acknowledge with thanks WAQF and the Deanship of Scientific Research (DSR) for technical and financial support. The authors would like to acknowledge the Deanship of Scientific Research, Vice Presidency for Graduate Studies and Scientific Research, King Faisal University, Saudi Arabia (Proposal number KFU263777).

The original version of this article has been updated.

